# Exploratory Analysis of Social Networks Linked to the Provision of Beverages in Costa Rican Schools

**DOI:** 10.3390/nu15102271

**Published:** 2023-05-11

**Authors:** Rafael Monge-Rojas, Rulamán Vargas-Quesada, Travis Moore, Christina D. Economos, Uriyoán Colón-Ramos

**Affiliations:** 1 Nutrition and Health Unit, Costa Rican Institute for Research and Education on Nutrition and Health (INCIENSA), Ministry of Health, Tres Ríos 4-2250, Costa Rica; rmonge@inciensa.sa.cr (R.M.-R.); rvargas@inciensa.sa.cr (R.V.-Q.); 2 Friedman School of Nutrition Science and Policy, Tufts University, Boston, MA 02155, USA; travis.moore@tufts.edu (T.M.); christina.economos@tufts.edu (C.D.E.); 3 Department of Global Health, Milken Institute School of Public Health, George Washington University, Washington, DC 20052, USA

**Keywords:** social network analysis, stakeholders, beverages, school environment, adolescents, Costa Rica

## Abstract

Sugar-sweetened beverages (SSBs) are implicated in weight gain and adverse cardiometabolic heath. Social networks of stakeholders involved in providing potable water and sugar-sweetened beverages (SSBs) in high schools in Costa Rica were studied using social analysis network. In public and private schools, the interactions between the stakeholders in charge of providing beverages are fragmented and their role in preventing the availability of SSBs is weak. School canteen owners ultimately decide what beverages are available at school, which may cause students to choose beverages that increase the risk of overweight/obesity. It is therefore urgently necessary to improve the capacity for two-way interactions between the stakeholders to enhance their roles in the provision of beverages. Hence, it is essential to reinforce the stakeholders’ leadership, and set up innovative ways to exert it in order to develop a shared vision of the types of drinks that should be available in the school environment.

## 1. Introduction

A high consumption of sugar-sweetened beverages (SSBs) increases the risk of obesity in adolescents [1,2,3], which in turn increases over time the prevalence of cardiometabolic risk factors and the risk of mortality [4,5,6]. About 70% of Costa Rican adolescents consume >15% of total energy intake (TEI) from added sugar (well above the WHO recommendation of <10%) [7] which is a risk factor in the rising prevalence of obesity among adolescents in Latin America [8,9].

To curb SSBs and added sugar consumption among schoolchildren and adolescents, the Costa Rican Ministry of Public Education issued a decree in 2012 to regulate the sales of SSBs and food on public school premises [10]. The food industry accepted this decree and began a reformulation of products to comply with the new regulations. They have not used tactics to obstruct the implementation of the decree [11]. In addition, before starting to manage school canteens, owners must sign a statement in which they agree to comply with the provisions of the decree [10].

The decree states that each school should establish a Health and Nutrition Committee that, together with the school principal and board, should oversee compliance with this regulation [10,12]. In addition, since 2017, the free school lunch menus in public schools were mandated to replace frescos (traditional homemade beverages made with fresh fruit juice, sugar, and water) with tap water [12]. However, a 2021 investigation revealed that SSBs are still available in public schools in Costa Rica [13], raising the question about the impediments to implement this school mandate.

Social network analysis (SNA) has been used to identify, map, and analyze the structure, composition, and patterns of interaction between all actors/stakeholders in a particular social system [14,15,16,17,18]. Understanding who the stakeholders are and how they interact can provide insight into how mandates are adopted, implemented, and sustained [19] because it allows for the visualization of discussion networks and the means with which they can be strengthened and leveraged to promote a healthy school food environment. The objective of this study was to: (1) systematically identify key stakeholders involved in the provision of all beverages (including potable drinking water) in schools in San Jose, Costa Rica; and (2) use social network analysis to explore and describe the beverage discussion networks within and between schools.

## 2. Materials and Methods

### 2.1. Study Design

This is an observational study that collected primary data with regard to stakeholders involved in the discussion networks’ provision of beverages in high schools in San Jose, Costa Rica. Data were collected over two months in 2022 via three sequential waves of data collection to arrive at the final discussion networks or the networks that consist of stakeholders who discuss the purchase or provision of beverages in their school. Private schools were added to the data collection to compare results with public schools.

### 2.2. Background and Setting

Data were collected from public and private urban high schools in San José, the capital of Costa Rica. This city includes 6.3% of the high school students in the country’s educational system, 50.2% of which are boys and 49.8% are girls. In general, 80% attend public schools and 20% attend private schools [20]. Students in public schools mainly belong to the first 3 income quintiles, while in private schools the majority of the students belong to income quintiles 4 and 5 [21].

### 2.3. Data Collection Survey Design

We adapted the network dimension portion of the stakeholder-driven community diffusion survey, which has been implemented in the United States with acceptable reliability to assess social and work network properties [22,23]. Given the objective of exploring beverage discussion networks across schools, the original survey went through several revisions and adaptations before administration. First, the survey was adapted by asking about the provision of beverages in each school; second, it was translated to Spanish by a Spanish native speaker from Costa Rica and revised by the bilingual research team; third, it was pilot-tested for comprehension with two school principals who did not participate in the study.

The survey was designed to start with school principals (person In charge of providing strategic direction for the school, hereafter referred to as ‘ego’) and continue with the people named by the ego (alters) as those with whom the provision of beverages in the school environment was discussed. Subsequently, the survey was continued with the people mentioned by the alters (the alters’ ties) and so on. The survey was concluded when the beverage suppliers were mentioned by some alters.

Each interviewee was asked about their sex, the type of school they worked in (private/public), and their function in the school (e.g., school principal, member of the School Board, member of the Health and Nutrition Committee, school canteen owner). Then, they were asked to name up to 20 individuals with whom they discussed to provide water (tap, bottled, etc.) and a list of beverages. The list came originally from the Beverage Intake Questionnaire (BEVQ-15) [24] on typically consumed beverages and was adapted by Colon-Ramos [25] to include examples from common Hispanic beverages. This list was reviewed by Costa Rican researchers (RM-R, RV-Q) to obtain the final list, which contained typically consumed beverages by adolescents in this country [7].

**Figure 1:**
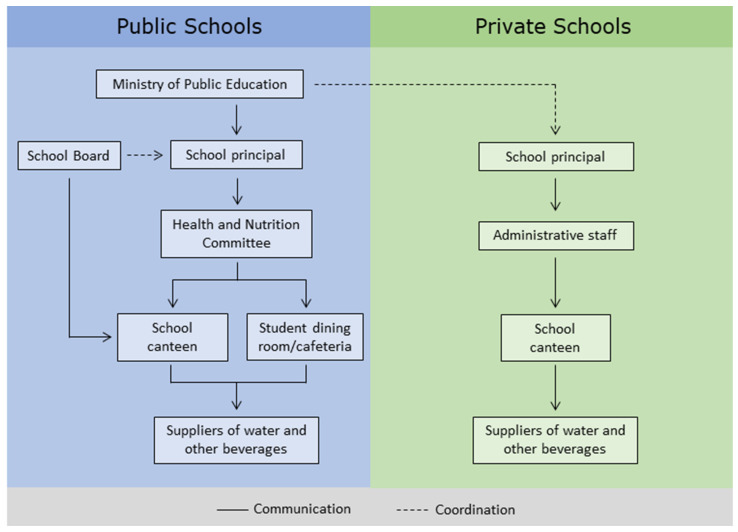
Sectors of the stakeholders in charge of the provision of beverages in a school environment according to type of school.

For each person named, the respondent was asked to select the following from a close-ended list: (1) the type of beverage (“When you interact with this person, which of the following beverages do you interact about? (choose all that apply: tap water, bottled water, sugar-sweetened soft drinks, diet/light soft drinks, prepackaged 100% fruit juices, prepackaged sugar-sweetened fruit-flavored still drinks, prepackaged fruit nectars, frescos, sports or rehydration drinks, energy drinks)”); (2) the sector (“Which sector does this person belong to? (choose one): school principal/administrative staff, School Board, Health and Nutrition Committee, school canteen, student dining room/cafeteria, beverage supplier, supplier of drinking water dispenser, Aqueducts and Sewers Services, local government, Ministry of Public Education”) (Figure 1 shows the sectors of the stakeholders according to type of school); (3) the frequency with which they interacted (“How often do you interact with this person? Choose one: daily, weekly, monthly, quarterly, yearly”); (4) the closeness (“How close do you feel to this person? 5-point Likert scale: 1—not close to 5—very close”) (“closeness”); and (5) how influential they thought this person was regarding the provision of beverages within their school (5-point Likert scale: 1—not influential to 5—very influential) (“influence”).

### 2.4. School Sampling and Recruitment

Public and private high schools in the area of San Jose, Costa Rica were selected using simple random sampling [26] from the 2020 schools’ roster of the Ministry of Public Education [27] and contacted sequentially to participate in the study.

Three public and three private schools were selected with the sole purpose of obtaining an initial understanding of the interactions between the stakeholders linked to the provision of beverages in the school environment. If that number of schools was insufficient to adequately understand the organizational hierarchy of the social networks within public and private schools, it would be increased until the goal was achieved. Selecting a representative sample of public and private schools in the area of San Jose was not intended at any time.

A trained research assistant first contacted the principals in each school via phone or email to explain the study objectives and protocols, and to invite them to participate using an approved recruitment script. If they agreed to participate, a date was selected to administer the survey in person. No monetary or other form of compensation was provided as an incentive to participate, in compliance with the research law in Costa Rica [28].

### 2.5. Data Collection

Data were collected in three sequential waves, starting with the ‘ego’ stakeholder (the school principal) and following up with the second-degree ‘alters’ (the persons named in the survey completed by each ‘ego’). The number of participants and their characteristics (e.g., sex, age) varied in each wave, since this depended on the relationship that each alter had with others alters (different actors linked to the provision of beverages in the school setting). Three waves of data collection were conducted. The third wave yielded a complete description of the network linked to the provision of beverages as respondents nominated already-named stakeholders and also the beverage suppliers. The same situation occurred in the six schools; therefore, it was not necessary to carry out additional waves to complete the description of the social network.

The duration of each round varied from 1 to 4 days based on the availability of the alters mentioned by the ego or the alters mentioned by other alters. Data collection lasted two months as the start of each wave depended on the availability of school principals to meet with the researchers. Data were collected simultaneously in public and private schools, and the order in which each school was added to the study depended solely on the availability of the school principal (the ego actor).

All surveys were administered in person in Spanish by a trained, bilingual researcher. In Wave 1, data were collected using the Qualtrics survey platform, and for waves 2 and 3, the paper survey was used, and data were then entered into the Qualtrics platform. The survey lasted approximately 15–20 min to complete.

Ethical considerations: The study protocol was approved by the Bioethics Committee of the Costa Rican Institute for Research and Education on Nutrition and Health (INCIENSA) under number IC-2022-01.

### 2.6. Data Analysis

Collected data constitute 100% of the stakeholders that make up the social networks linked to the provision of beverages in public and private school settings. Therefore, regardless of the number of participants, the use of social network analysis is an appropriate methodology for this study, based on the premise that a social network consists of a finite set of actors and the relationships, or ties, defined on them [29,30].

Data collected from all three waves were combined to form a social network within each school using iGraph (a package that allows graphing the interconnections (edges) between individuals or nodes (vertex) that make up the social network) [31] and Statnet (a collection of packages for the statistical computing system that supports the representation, manipulation, visualization, modeling, simulation, and analysis of relational data) [32] in R statistical software [33].

We used the following scenarios to create a directed network: (1) stakeholders A and B both responded to the survey and nominated each other (counts as two ties); (2) A and B responded, but A nominated B or B nominated A (counts as one tie); and (3) only A or B responded and nominated the other (counts as one tie). Once the directed networks were created, we ran a series of analyses, both descriptive and exploratory, to examine the presence of network and ego patterns within and across schools.

First, descriptive analyses were performed on each school’s network to examine both network and ‘ego’ metrics. These metrics included density, centrality (degree, closeness, and betweenness), and homophily (i.e., degree and nominal assortativity). Network density is the measure of the number of ties within the network. Degree centrality is the number of ties an individual stakeholder (i.e., an ‘ego’ or an ‘alter’) has. Closeness centrality measures the distance each individual stakeholder is from all other stakeholders. Betweenness centrality measures how many times an individual lies on the shortest path between two other individual stakeholders.

Second, triads (or groups of three) and tetrads (groups of four) were assessed following this supposition: the tendency for individual i to be tied to individual k if a tie exists between individual i and individual j and between individual j and individual k. Identifying triads and tetrads in a network can be used to determine whether and why there is hierarchy, if that hierarchy is linked to transitivity (i.e., the tendency toward clustering), which can be linked to how information flows through a discussion network. Hierarchy in networks emerges from one-way ties. In other words, the directionality of an unreciprocated tie indicates a hierarchical difference, where the recipient of a tie occupies a higher position than the sender.

Third, we built visualizations based on the reported frequency of contact and perceived stakeholder influence, as indicated by the thickness of each tie. To highlight differences, the thickness of the connection was scaled between 1 and 20 times the reported frequency (range: 1–5 points (pts); average: 2.89 pts) and influence (range: 1–5 pts; average: 2.58 pts) metrics. Thus, a low connection thickness represented a lower score and a high connection thickness represented a higher score. Generally, a high frequency of contact and a high perceived influence refer to scores of 4 pts and above, which corresponds to be involved in the provision of beverages weekly or more and rating a stakeholder as influential or highly influential, respectively. These visualizations also highlighted other raw data such as the different drinks that were discussed between stakeholders and each stakeholder’s sector or function within the school. These visualizations aided in examining whether there were any links between the frequency of contact, stakeholder influence, and hierarchy.

Finally, we utilized the Clauset–Newman–Moore algorithm to examine community clustering to identify whether specific clusters or groups existed within schools and whether these clusters were based on sector affiliation or how stakeholders felt about each other (e.g., close, influential).

## 3. Results

Data were collected from six schools (three public and three private). Table 1 summarizes the characteristics of all stakeholders who were mentioned as involved in the social network linked with the provision of beverages in each type of school. The food industry was not mentioned as an influential stakeholder in beverage provision across public nor private schools. The number of stakeholders tends to be higher in public schools, since their social networks are more complex than those in private schools, as will be described later. This table highlights the presence of 15 beverage suppliers in a private school and the participation of stakeholders from various sectors in public schools that are not present in the private ones. Some characteristics of the stakeholders (e.g., gender and mean age) vary across schools due to the peculiarities of each social network.

**Figure 2:**
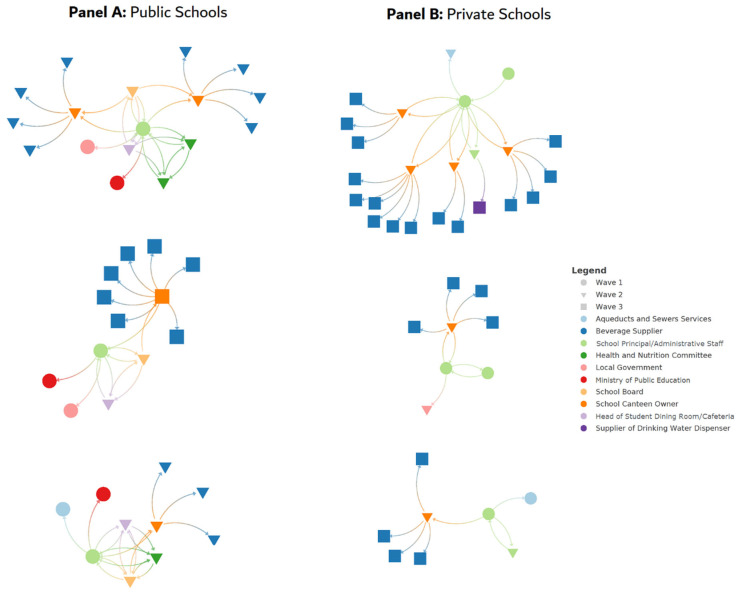
Network ties in each public (**Panel A**) and private school (**Panel B**) regarding beverage provision in their schools and by data collection wave. Note: Different shapes (circle, triangle, and square) represent waves of data collection. Colors represent different sectors (e.g., red = Ministry of Public Education) and different functions (e.g., orange = school canteen owner) within each beverage discussion network. Each arrow indicates the directionality of the interaction between stakeholders, which can be unidirectional (only from one stakeholder to another) or bidirectional (from one stakeholder to another and vice versa).

The ties and direction of connections between all stakeholders are illustrated by each school in Figure 2.

Private School Beverage Discussion Networks: In private schools, the school principals, but mainly administrative staff (staff who assist in school routines, classroom activities, financial and administrative matters, and in developing school policies), are directly connected to school canteen owners to discuss the types of beverages they would make available in their schools. In two private schools, this tie was bidirectional (school canteen owners also reported the administrative staff as part of their beverage discussion network). Table 2 shows the variety of beverages offered in private schools compared to public.

Public School Beverage Discussion Networks: In public schools, the ‘egos’ (i.e., school principals) primarily interacted with three entities regarding the provision of beverages in their schools: their School Board, their school’s Health and Nutrition Committee, and the school cafeteria. These interactions were bidirectional/reciprocal, meaning that these ‘alters’ also mentioned interacting with school principals regarding the provision of beverages. In terms of the types of beverages, these ‘egos’ and ‘alters’ reported that they discussed exclusively the provision of water (bottled or tap). School principals also interacted with the municipal water authorities regarding tap water in their schools.

The School Board also interacted with the school cafeteria and with the school canteen owners to ensure only the provision of water (tap or bottled) in schools. However, school canteen owners did not name the School Board as part of their network in the provision of beverages. School canteen owners only named beverage suppliers as stakeholders with whom they discussed beverage provision in schools. In addition to bottled water, school canteen owners discussed up to eight different types of SSBs with beverage suppliers for public schools (Table 2). None of the school canteen owners named prepackaged energy drinks because their sale in the school environment is prohibited by the Ministry of Public Education [10], and school principals strictly apply this institutional guideline.

The ties and networks resulting from the Health and Nutrition Committees at each public school were more equivocal: in one school, this committee was not mentioned at all as part of the school principal or School Board’s network regarding the provision of beverages in their school. In the other two public schools where this committee was mentioned, it was tied with school cafeteria personnel either bidirectionally or unidirectionally (the latter meaning that the school cafeteria personnel did not mention the committee in their network). In either case, the discussions were exclusively about the provision of tap water in schools.

### Level of Influence in Beverage Discussion Networks

**Figure 3:**
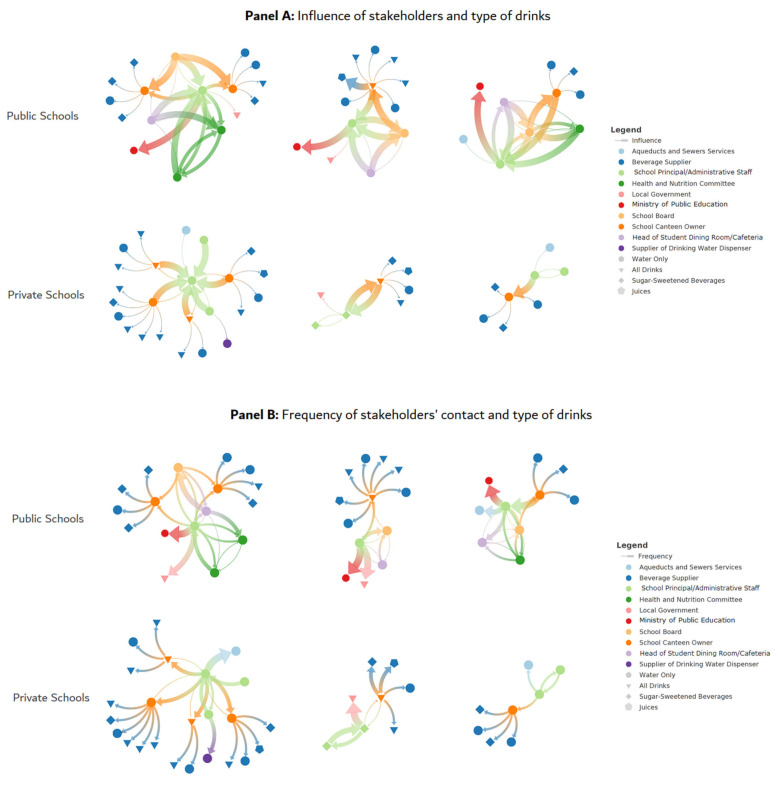
Influence, contact frequency, and drink type within beverage discussion networks. Note: (**Panel A**) illustrates stakeholders’ reports of how influential their ties were in discussing the purchase and provision of beverages within their school. (**Panel B**) illustrates stakeholders’ reports of how frequently stakeholders discussed the purchase and provision of beverages with other stakeholders within their school. Different tie thickness represents varying levels of either influence or frequency, depending on the figure (the thicker the tie the more influence or the more frequent the contact compared to other stakeholders in the network). Different colors represent sectors, as seen in Figure 1. Shapes represent the types of beverages that were discussed with the respective stakeholder. Private schools are depicted on the bottom half of each figure, and public schools are depicted on the top half of each figure.

The level of influence on beverage provision within each school stakeholder network and the frequency with which stakeholders interacted was based primarily on two factors: school type and the organizational position of the stakeholder (Figure 3).

Level of influence in private schools: School principals and administrative staff identified school canteen owners as frequent contacts with high influence on the purchase and provision of beverages in their schools. An exception to this trend is found in one private school where the administrative staff has a higher level of influence on the purchase and provision of bottled water and SSBs than the school canteen owners (3.71 pts vs. 3.12 pts, respectively).

Level of influence in public schools: In public schools, school principals named the Ministry of Public Education as a stakeholder with high influence (ranked 5 pts on a scale of 1–5 pts) in shaping their understanding of the purchase and provision of beverages in schools. In contrast, other stakeholders in public schools, mainly in the School Board, had a higher frequency of contact with school canteen owners and tended to rate them as highly influential in the purchase and provision of only bottled water, even though school canteen owners (low-influence stakeholders) discussed that they offered a wide variety of SSBs in addition to bottled water. The Health and Nutrition Committee was perceived to have a low influence on the provision of water in school cafeterias (2.12 pts). Finally, school principals were identified by five types of stakeholders as being more influential regarding the provision of beverages compared to other stakeholders within the network (i.e., School Board, Health and Nutrition Committee, administrative staff), although they remained as having a lower frequency of contact (2.32 pts on average).

Network metrics displayed in Table 3 suggest a local hierarchy in public schools between school principals, the School Board, and the Health and Nutrition Committee as evidenced by the number of transitive triads (n = 6) and tetrads (n = 4) (Table 3). These same subnetworks also display higher levels (e.g., 4.23 pts) of influence among stakeholders, and, comparatively, higher levels of frequency of contact (e.g., 4.19 pts). Thus, stakeholders in higher positions in the hierarchy within schools (e.g., school principals and School Board members) tend to form clusters and have higher levels of influence on stakeholders in lower positions (e.g., school canteen owners).

Comparing metrics, public schools had a higher degree of centrality (i.e., number of ties that a stakeholder has) suggesting that stakeholders in public schools tend to be local connectors but are not necessarily connected to the wider network (e.g., school beverage suppliers).

In summary, beverage discussion networks in public schools were more interconnected compared to private schools, where it was often the school principal/administrative staff that connected directly with the beverage supplier. In the beverage discussion networks of public schools, water was discussed more than any other type of beverage among stakeholders, but school canteen owners discussed all other beverages, including SSBs. By contrast, in private schools, all types of beverages, including water and SSBs, were discussed between the ‘egos’ (school principals/administrative staff) and the school canteen owners directly.

## 4. Discussion

The youth spend a significant amount of their lives in schools and around their classmates [34]. The school environment and the activities that take place there can shape the youths’ preferences and behaviors, both by peer influence and role modeling of the authority figures at school [35,36,37]. This led the Ministry of Public Education in Costa Rica to enact a 2012 mandate banning beverages with >6 g of sugar/100 mL from school canteens in public schools [10], declaring that potable drinking water in school cafeterias should instead be provided. To help implement this mandate, the Ministry asked public schools to create School Boards and Health and Nutrition Committees. Our results show that the beverage discussion networks in public schools were hierarchical (from the school principal to the School Board, and to the Health and Nutrition Committee to student dining rooms/cafeterias and school canteens). Discussion networks that involved school administration focused exclusively on tap and bottled water. Although school canteens were mentioned in this discussion network, owners of these canteens did not mention the school principals, School Boards, or Health and Nutrition Committees as part of their beverage discussion networks. Instead, their networks were directly tied to the beverage industry, discussing a wide variety of SSBs provision in addition to bottled water to make available to public school students. Therefore, information on tap and bottled water flowed from school principals and administrative staff to canteens, but there was no flow of information from school canteens to the school principal, the School Board, or the Health and Nutrition Committee. These findings underscore opportunities for strengthening the structures of beverage discussion networks in public schools in Costa Rica. A coregulation approach might be very beneficial: school principals would not only delegate the provision of drinks to other stakeholders, but also become involved in monitoring which drinks are available in student dining rooms/cafeterias and school canteens. Since school principals are identified as the most influential stakeholders in the network, their supervisory powers carry a lot of weight. It is also urgent to improve the capacity for two-way interactions between the various actors to enhance their roles in the provision of beverages. Hence, it is essential to develop a shared vision of the types of drinks that should be available in the school environment, reinforce the stakeholders’ leadership, and set up innovative ways to exert it. This is particularly relevant if the power that school canteen owners currently have over the provision of beverages in the school setting is to be reduced.

According to a recent systematic review, legislative/environmental interventions were more effective than educational/behavioral interventions at decreasing SSBs consumption among adolescents, highlighting the power of well-implemented regulatory mandates to start lowering SSBs consumption in schools [38]. However, to assess the impact of regulatory policies, it is necessary to first continuously monitor whether schools are complying with the regulation [39,40]. Otherwise, it would be necessary to carry out implementation research to assess the feasibility, adoption, and acceptance of the regulation, as well as its coverage, quality, equity, efficiency, scale, and sustainability [41]. This can be achieved through stakeholder feedback, constraints reduction plans, initial and continuous adaptation of the strategy to the local context, a broad-based support of stakeholders, and coordination and educational community organization [41].

In Costa Rica, despite the national mandate, SSBs are still available in public school settings [13], indicating a disconnect between practices which schools are mandated to perform and that which they are instead conducting with regard to SSBs beverages [10]. Our findings from three public schools in Costa Rica suggest that the reason for this disconnect may lie in fragmented beverage discussion networks and a lack of feedback loops. For instance, for drinking water (tap and bottled), this network is hierarchical and it is unidirectional from the school principal, the School Board, and the Health and Nutrition Committee to school canteens. The next step would be to verify that bottled water is indeed made available in school canteens, or whether a feedback loop regarding the feasibility of this mandate would be needed. In contrast, for SSBs and other beverages (which are not allowed in school premises), these discussions are disconnected from the school principal, School Board, and Health and Nutrition Committee network and occur directly and exclusively between canteen owners and beverage suppliers. Here, one potential explanation is that the information regarding which beverages are allowed in school premises is not being monitored and supervised. Encouraging feedback loops from school canteens to other school stakeholders could further inform the reason behind this disconnect in SSBs availability.

While the networks are simpler in private schools, particularly the influence of the administrative staff, it is insufficient to guarantee an adequate provision of healthy beverages to students. A more active role of the school principal in the monitoring of beverages provision, through a greater interaction with school canteen owners, could contribute to changing the picture, and provide a better eating environment in private schools.

Given the actors’ deficient exercise of power in public and private schools, school canteen owners ultimately decide what beverages are available at school, which may cause students to choose beverages that increase the risk of overweight/obesity [34]. This requires the immediate attention of the Ministry of Public Education’s respective departments in order to promote healthy eating habits in public and private schools.

The results of this study should be interpreted from the perspective of its limitations and strengths. First, the study only included three public and three private schools, so the results cannot be generalized to the entire city of San Jose or the country at large. Nevertheless, the present work is a valuable addition to the scarce literature on networks involved in the provision of beverages in the school environment in Latin America. Second, participating schools were in urban areas, so the type of stakeholder networks and their interactions may differ from those in rural areas. Third, the study is cross-sectional and does not allow the identification of changes in the stakeholders that make up social networks or in the changes that occur in the interaction between them. Fourth, the respondents could have overestimated the assessment of their influence or that of other stakeholders due to their subjective perception since no objective measure was used to determine or confirm it. However, the SNA has become a theoretical, methodological, and technical alternative, which has resulted in new possibilities for social research. Fifth, our final discussion networks only accurately represent the organizational hierarchy within public and private schools included in our analyses. Finally, although this study included some artificially sweetened soft drinks (e.g., diet/diet sodas), it did not perform an in-depth analysis of the availability of this type of beverage in the school setting. Given the deleterious repercussions for health associated with the consumption of artificially sweetened soft drinks [42], their availability in the school environment should be considered in future studies.

## 5. Conclusions

In public and private schools, the interactions between the stakeholders in charge of providing beverages are fragmented and their role in preventing the availability of SSBs is weak and depends on decisions made by school canteen owners. This was evidenced by the SNA, which provided information on the existing stakeholders and institutional relationships, the existing decision framework and, therefore, the influence and exchange of information to achieve a school environment where healthy beverages are available.

It is necessary to develop public health policies aimed at preventing non-communicable diseases from adolescence and complying with the third sustainable development goal, which indicates the urgency of guaranteeing a healthy life and promoting well-being for all at all ages [43]. The adequate implementation in Costa Rica of the existing regulatory framework for beverages in school settings could contribute to the achievement of this objective. In addition, the use of front labels on prepackaged foods would make an important contribution to educating adolescents on making informed decisions when choosing the beverages they want to consume in the school setting. This could put pressure on school stakeholders by refocusing the availability of beverages in the school environment and improving compliance with the regulations issued by the Ministry of Public Education.

## Figures and Tables

**Table 1:** Characteristics of stakeholders involved in the provision of beverages in three public and three private high schools in San Jose, Costa Rica.

Stakeholder Characteristics	Public Schools			Private Schools		
	School 1 (n = 17) ^2^	School 2 (n = 10) ^2^	School 3 (n = 13) ^2^	School 1 (n = 24) ^2^	School 2 (n = 8) ^2^	School 3 (n = 8) ^2^
Gender (%) ^1^						
Female	29	40	50	86	67	0
Male	71	60	50	14	33	100
Age (y, mean)	54	31	43	46	38	45
Sector/Function (n)						
School principal/administrative staff	1	1	1	3	2	2
School Board	1	1	1	0	0	0
Health and Nutrition Committee	2	1	0	0	0	0
Head of student dining room/cafeteria	1	1	1	0	0	0
School canteen owner	2	1	1	4	1	1
Beverage supplier	8	3	7	15	4	4
Supplier of drinking water dispenser	0	0	0	1	0	0
Aqueducts and Sewers Services	0	1	0	1	0	1
Local government	1	0	1	0	1	0
Ministry of Public Education	1	1	1	0	0	0

^1^ Gender of those stakeholders who answered the survey: Public 1 = 7 people; Public 2 = 5 people; Public 3 = 4 people; Private 1 = 8 people; Private 2 = 3 people; Private 3 = 3 people. ^2^ Number of stakeholders mentioned in the network of beverage provision per school.

**Table 2:** Types of available beverages sold in the school canteens by type of school.

Type of Beverages	Public Schools			Private Schools		
	School 1	School 2	School 3	School 1	School 2	School 3
Bottled water	✓ ^1^	✓	✓	✓	✓	✓
Sugar-sweetened sodas	✓	NA ^2^	NA	✓	✓	NA
Diet/light sodas	✓	NA	NA	✓	✓	✓
Prepackaged 100% fruit juices	✓	✓	✓	✓	✓	✓
Prepackaged fruit-flavored beverages with added sugars	✓	✓	✓	✓	✓	✓
Prepackaged fruit nectars with added sugars	✓	✓	✓	✓	✓	✓
Frescos (fruit-flavored beverages with added sugar prepared in schools)	✓	NA	✓	✓	✓	✓
Prepackaged sports or rehydration drinks	✓	NA	NA	✓	✓	✓
Prepackaged energy drinks	NA	NA	NA	NA	NA	NA

^1^ ✓: Beverage available in school canteens. ^2^ NA: Beverage not available, according to information provided by school canteen owners.

**Table 3:** Comparison of social network metrics across public and private schools.

Type of Beverages	Public Schools			Private Schools		
	School 1	School 2	School 3	School 1	School 2	School 3
Density	0.1	0.12	0.21	0.05	0.19	0.13
Degree centrality	3.29	2.67	3.8	2.35	2.29	1.75
Connections	28	16	19	27	8	7
Closeness centrality	0.482	0.636	0.678	0.491	0.694	0.643
Reciprocity	0.27	0.23	0.46	0.23	0.33	0.00
Betweenness centrality	0.07	0.121	0.162	0.064	0.165	0.095
Transitive triads	3	2	2	1	0	0
Transitive tetrads	2	1	1	0	0	0
Primary drink ^1^	Water	Water	Water	All beverages	SSBs	Water

^1^ Indicates which drink was named the most often in the school discussion network. Note: Metrics here are meant to guide comparison across schools and are not informative in themselves. For example, Public school 1’s degree of centrality is higher than Private school 1’s degree of centrality, a comparison that can aid in exploring differences in networks based on the type of school. Density is the measure of the number of ties within the network. Degree centrality is the number of ties an individual stakeholder has. Closeness centrality measures the distance each individual stakeholder is from all other stakeholders. Reciprocity of the network indicates how many ties are reciprocated; a reciprocity of 1 would mean all stakeholders nominated those who nominated them. Betweenness centrality measures how many times an individual lies on the shortest path between two other individual stakeholders.

## Data Availability

The data presented in this study are available on request to the corresponding author.
